# Rotation-traction manipulation induced intradiskal pressure changes in cervical spine—an *in vitro* study

**DOI:** 10.3389/fbioe.2024.1322212

**Published:** 2024-02-08

**Authors:** Changxiao Han, Minshan Feng, Haibao Wen, Xunlu Yin, Jing Li, Wuyin Du, Bochen Peng, Guangwei Liu, Liguo Zhu

**Affiliations:** ^1^ Wangjing Hospital of China Academy of Chinese Medical Sciences, Beijing, China; ^2^ Key Laboratory of Beijing of TCM Bone Setting, Beijing, China; ^3^ Graduate Studies of Beijing University of Chinese Medicine, Beijing, China

**Keywords:** rotation-traction manipulation, intradiskal pressure, cervical intervertebral discs, biomechanics, cervical spine traction

## Abstract

**Objective:** Evaluate the effect of rotation-traction manipulation on intradiskal pressure in human cervical spine specimen with different force and duration parameters, and compare the intradiskal pressure changes between rotation-traction manipulation and traction.

**Methods:** Seven human cervical spine specimens were included in this study. The intradiskal pressure was measured by miniature pressure sensor implanting in the nucleus pulposus. rotation-traction manipulation and cervical spine traction were simulated using the MTS biomechanical machine. Varied thrust forces (50N, 150N, and 250N) and durations (0.05 s, 0.1 s, and 0.15 s) were applied during rotation-traction manipulation with Intradiscal pressure recorded in the neutral position, rotation-anteflexion position, preloading, and thrusting phases. Futuremore, we documented changes in intradiscal pressure during cervical spine traction with different loading forces (50N, 150N, and 250N). And a comparative analysis was performed to discern the impact on intradiscal pressure between manipulation and traction.

**Results:** Manipulation application induced a significant reduction in intradiscal pressure during preloading and thrusting phases for each cervical intervertebral disc (*p* < 0.05). When adjusting thrust parameters, a discernible decrease in intradiscal pressure was observed with increasing thrust force, and the variations between different thrust forces were statistically significant (*p* < 0.05). Conversely, changes in duration did not yield a significant impact on intradiscal pressure (*p* > 0.05). Additionally, after traction with varying loading forces (50N, 150N, 250N), a noteworthy decrease in intradiscal pressure was observed (*p* < 0.05). And a comparative analysis revealed that rotation-traction manipulation more markedly reduced intradiscal pressure compared to traction alone (*p* < 0.05).

**Conclusion:** Both rotation-traction manipulation and cervical spine traction can reduce intradiscal pressure, exhibiting a positive correlation with force. Notably, manipulation elicits more pronounced and immediate decompression effect, contributing a potential biomechanical rationale for its therapeutic efficacy.

## 1 Introduction

Manual therapy interventions are a preferred treatment for both healthcare professionals (Ferrari and Russell, 2004; Poitras et al., 2005; Sherman et al., 2006; Carlesso et al., 2014)and patients with musculoskeletal pain conditions (Broom et al., 2012; Hurwitz, 2012; Aickin et al., 2013; Murthy et al., 2015). Spinal manipulation is one of the most commonly used manual techniques and is widely popular worldwide. It exerts therapeutic effects by applying high-velocity, low-amplitude forces to specific areas of the spine (Wirth et al., 2019), with demonstrated clinical efficacy in reducing muscle inhibition (Vining et al., 2020), modulating neuromuscular excitability (Kingett et al., 2019), and correcting proprioceptive deficits (Haavik et al., 2018).

Clarifying the therapeutic mechanisms inherent to manual therapy has consistently been a pivotal focus in specialized domain. Establishing the biological effects of treatment can assist clinicians in elucidating the mechanisms contributing to clinical populations and matching appropriate therapeutic measures (Clauw, 2015; Edwards et al., 2016). Mechanism-based manual therapy epitomizes a judicious and precisely targeted approach (Granovsky and Yarnitsky, 2013; Edwards et al., 2016). Bialosky and Pickar have proposed comprehensive models for the therapeutic mechanisms of manual therapy, suggesting an integrated interaction between biomechanics, neurophysiology, psychological factors, and the endocrine system (Pickar, 2002; Bialosky et al., 2008; Bialosky et al., 2018). Among these, biomechanical mechanisms are considered a necessary condition that initiates a cascade of neurophysiological and endocrine responses.

Biomechanical studies have extensively examined the effects of spinal manipulation on the spine, but they have provided limited insights into the underlying mechanisms responsible for the clinical efficacy it offers (Bialosky et al., 2018; Gyer et al., 2022). One aspect explored in these studies is the decompression of the intervertebral disc during spinal manipulation (Lisi et al., 2006; Mitchell et al., 2017). It has been suggested that this technique reduces joint surface loading and nerve root compression, potentially alleviating discogenic pain associated with disc degeneration. And the transient reduce in intradiskal pressure can trigger a series of subsequent reactions, including modulation of pain fiber conduction and adjustment of nociceptors. By relieving pressure, spinal manipulation offers a biomechanical explanation for the pain relief experienced by certain patients.

However, this biomechanical explanation have reported conflicting evidence. Maigne and Deursen and van Deursen DL et al. (Maigne and Guillon, 2000; Van Deursen et al., 2001a; Van Deursen et al., 2001b) Studies have shown a significant reduction in disc pressure after spinal manipulation, both in human and animal experiments. In constrast, Brenda study (Yantzer et al., 2007) shows that Small torsion torques has no significant difference in intradiscal pressures or disc heights. The study also suggested, in contrast to other studies, that purely axial torsional forces hardly change the Intradiskal pressure, while proper angular displacement (0.5°–2.0°) reduces the pressure. From a biomechanical perspective, axial rotation has minimal impact on intradiscal pressure, while an additional vertical force is applied that intradiscal pressure is further influenced.

Unlike traditional spinal manipulation, the Rotation-Traction Manipulation (RTM) changes thrust direction from basically rotation to longitudinal traction. Studies have shown that RTM can reduce the risk of excessive rotation through the subject’s active rotational position and the operator’s upward thrust (Zhu et al., 2017). Based on the knowledge of the manipulation effecting on intradiscal pressure and spinal biomechanics in previous studies, we believed that the longitudinal traction force of RTM is more helpful in reducing intradiscal pressure. However, to our knowledge, there are no biomechanical studies that provide above information in the class of spinal manipulation characterized by longitudinal traction thrust. And there is a lack of comparative studies investigating the differences in intradiscal pressure effects between manipulation and cervical traction.

**FIGURE 1:**
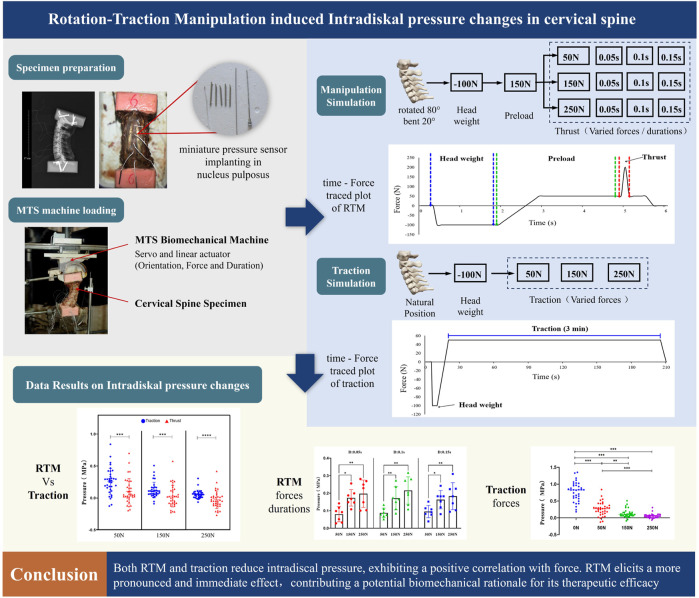
Graphical abstract.

Therefore, the purpose of this study was to evaluate the effect of different biomechanical parameters of RTM on intradiscal pressures in cervical spine specimens, and compare the differences in intradiscal pressures between manipulation and traction. We hope the results may provide a potential biomechanical explanation for the mechanisms of RTM Figure 1.

## 2 Methods

### 2.1 Specimen preparation

**FIGURE 2:**
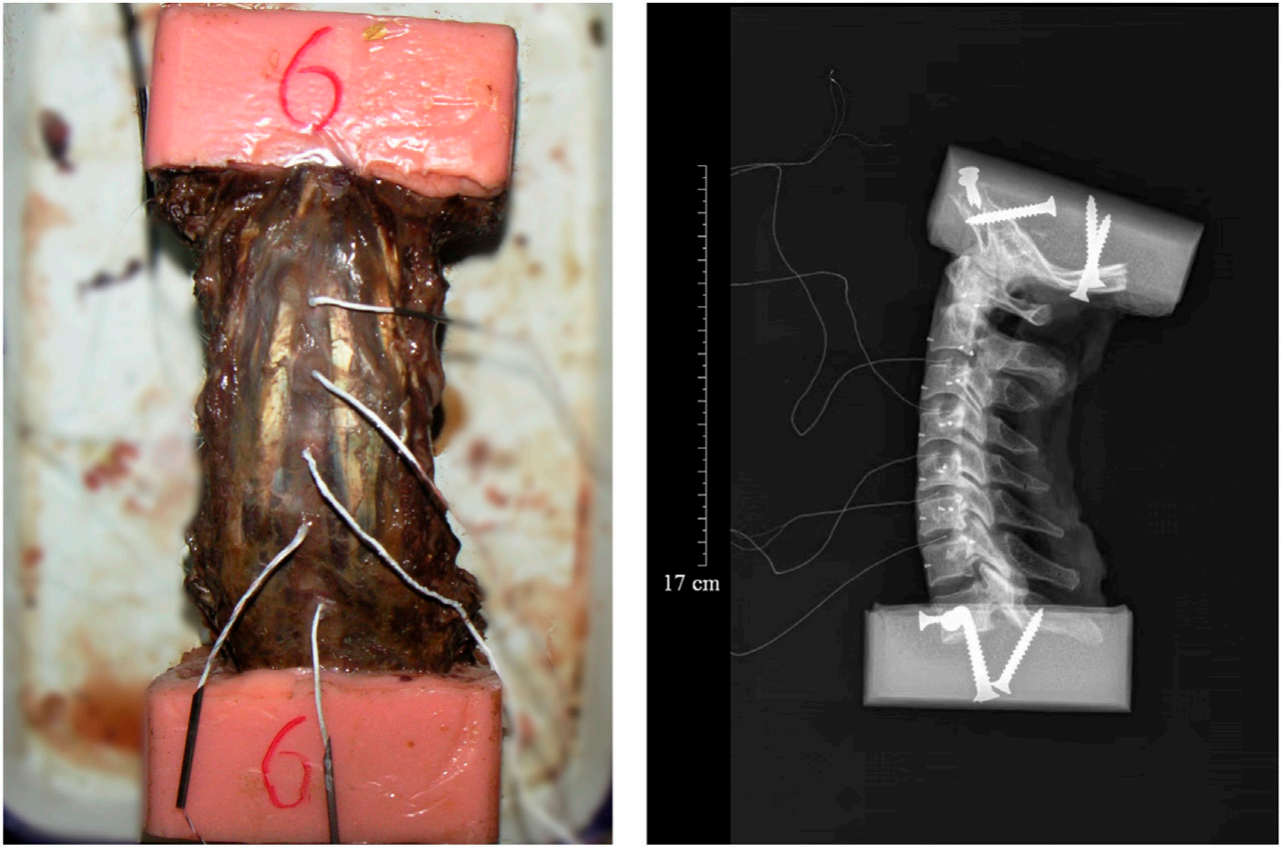
Cervical spine spicemen with the Miniature pressure sensor embedded in the nucleus pulposus (Left) and X-Ray of cervical spine spicemen (Right).

Seven adult male cervical spine specimens with a mean age of 39.3 ± 4.9 years were obtained for testing in this experiment (source of specimens was the Beijing Anatomical Society). All specimens were free of severe spinal deformities and history of cervical spine surgery. The upper end of the cervical spine specimens were dissected at 10 cm above the occipital foramen and at the level of the T1-2 disc, preserving the integrity of the cervical ligaments and small joints. The specimens were stored in a low-temperature refrigerator at −20°C and thawed naturally before testing. The upper cranial base and the lower T1 vertebrae were placed in plastic containers and embedded with polymethylmethacrylate, exposing only the cervical segment (C1-C7). The level of the upper and lower embedding blocks was not greater than 0.1°; the greater foramen of the occipital bone was parallel to the horizontal plane, and the upper edge of the C6 vertebral body was at an approximately 20° anterior angle to the horizontal (simulating a normal neutral spine position). Finally, the puncture needle was used to guide the micro-pressure sensitive element coated with silicone into the nucleus pulposum to measure the pressure. After pulling out the puncture needle, the air intake was sealed with 401 glue. Only the wire of the sensor was connected to the external recorder to keep the seal in the nucleus pulposum. As the nucleus pulposus belongs to gelatinous substances, whose inner state accords with Pascal law, therefore, the plantation direction of the sensors is not specially disposed. Radiographs were taken after all specimens were processed to confirm the location of the micropressure-sensitive elements (Figure 2).

### 2.2 Experimental equipment

(1) MTS Biomechanical Machine: Model MTS858.02, America. Minnesota. Company. (2) Miniature pressure sensor: Produced by precision measurement company of the United States, Model 060, measuring range of 0–1.4Mpa, diameter of 1.5mm, thickness of 0.3 mm.

### 2.3 Rotation-traction manipulation

**FIGURE 3:**
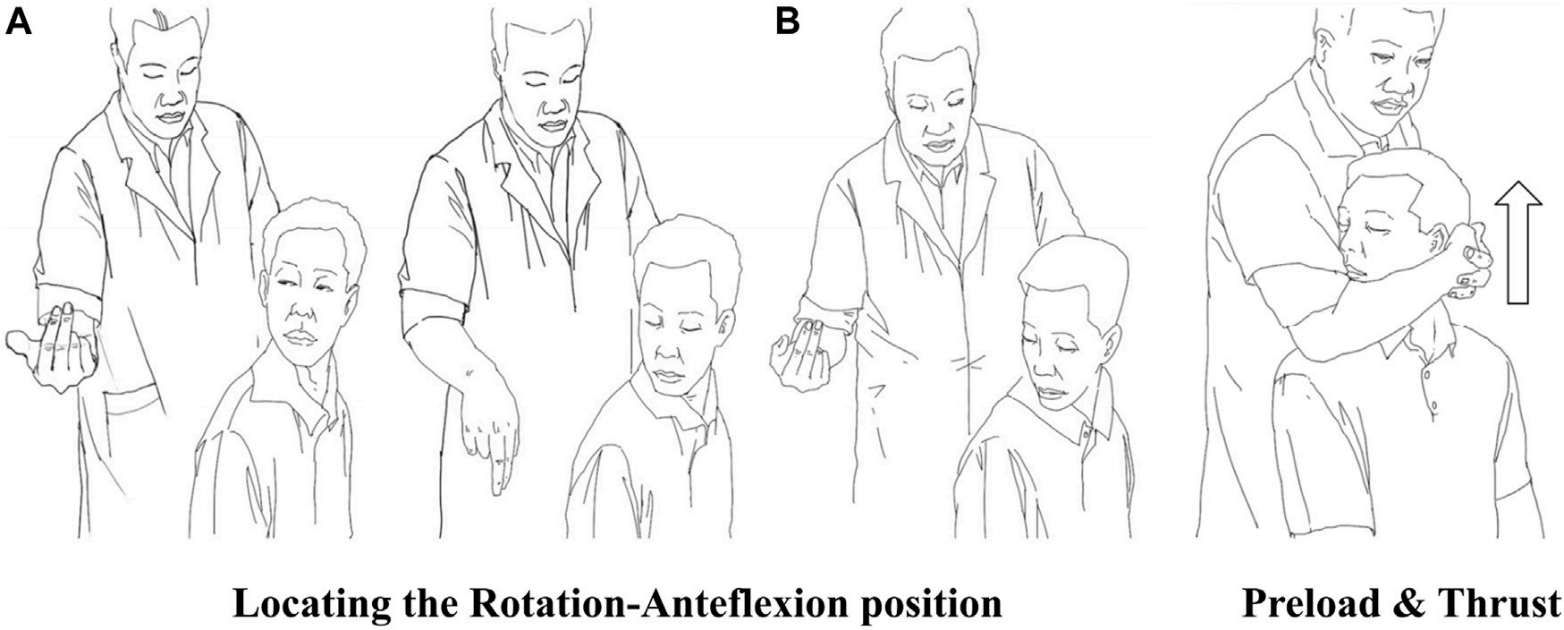
Procedure of RTM. **(A)** shows the subject’s active rotary-position process of rotation-flexion-rotation under guidance. **(B)** shows a physiotherapist performing preload traction and upward-thrust.

The subjects were manipulated in an upright seated position. The physiotherapists stood behind the subjects. Taking RTM of the right side as an example (Figure 3), the parameters were as follows: (1) rotation-Anteflexion position: the patient’s head was guided to rotate to the right direction limit, then flexed, and finally rotated to the right direction limit again. (2) Preload: the patient’s mandible was held in the manipulator’s forearm and then pulled slowly upward for about 3–5 s (3) upward-thrust: the head was thrust upward rapidly after pretraction and a “click” was always heard.

The preceding text outlines the clinical procedure for the RTM. In this study, we reproduce this technique through MTS Biomechanical machine simulation based on previously quantified biomechanical data (Huang et al., 2017; Zhu et al., 2017).

### 2.4 Experimental procedures

The experiment is to be carried out at the Key Laboratory of Beijing of TCM Bone Setting, and the room temperature during the experiment remains around 25°C. Fix the prepared specimens on the experimental biomechanical machine (MTS858) Which are Servo and linear actuator motor to ensure that cervical spine specimens are operated under predetermined conditions (direction, force and duration). Three 350 Ω resistors were connected to micropressure-sensitive elements to form a Wheatstone bridge, and the leads were connected to the data acquisition system to form a pressure measurement system to monitor the changes of Intradiskal Pressure with varis manipulations loaded by MTS machine.

RTM procedure: the following loading plan was designed based on the biomechanical data (Huang et al., 2017; Zhu et al., 2017): First, the traction force of the machine was zeroed out, and a force of 100N was preloaded according to the center of gravity of the head to simulate the weight of the head. Then, the cervical spine specimen was rotated 80° and bent forward 20° to reach the preset position of the cranking maneuver. First, it was slowly pulled upward with a force of 150N for 3 s to simulate the clinical preloading operation process. Then, it was quickly pulled upward with the force of 50N, 150N and 250N within 0.15 s, 0.1 s and 0.05 s respectively. The thrust process of different time periods and forces was simulated.

Cervical spine traction procedure: Firstly, the machine’s loading force was reset to zero, and a preload force of 100N was applied according to the head’s center of gravity to simulate the weight of the head. Subsequently, maintaining the cervical spine in a neutral position, forces of 50N, 150N, and 250N were individually applied in an upward direction. The forces were uniformly increased over a period of 10 s and then sustained for 3 min to emulate the clinical cervical spine traction process.

Two small load/unload cycles were performed before each formal loading to minimize viscoelastic effects on the cervical spine. The interval between each operation is 5 min to make the cervical vertebra creep deformation and ensure the stability of the experimental results.

### 2.5 Data analysis

Data were analysed using SPSS software. All statistical tests were conducted using bilateral tests, and if the *p*-value was less than or equal to 0.05, the differences tested would be considered statistically significant. Quantitative data are expressed as the Mean (SD). According to the research objectives and data characteristics of this study, paired *t*-test, One-way ANOVA and LSD tests were used were selected for statistical expression.

## 3 Results

### 3.1 Variation of intradiscal pressure during RTM

**FIGURE 4:**
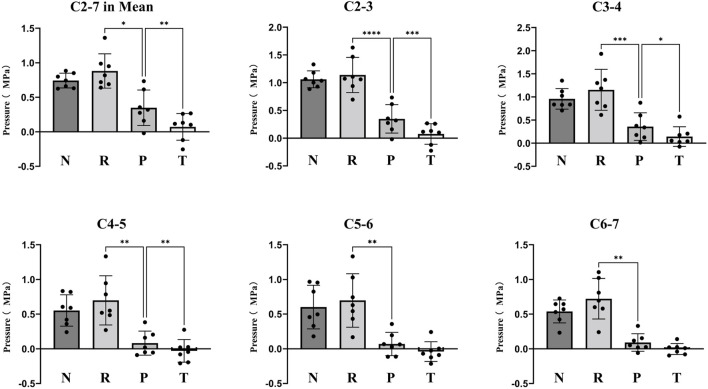
Comparison of Intradiscal pressure in different manipulation conditions. N: Neutral Position. R: Rotation-Anteflexion. P: Preload. T: Thrust.

The mean intradiscal pressures were 0.74 (0.31) MPa in the neutral position, 0.88 (0.40) MPa in the rotational-anterior flexion position, 0.19 (0.24) MPa in the preload, and 0.03 (0.17) MPa in the thrust for all five discs. There was a significant decrease of Intradiskal pressure during preloading and thrusting in every cervical intervertebral disc (*p* < 0.05), while the difference between thrust and preload for C5-6 and C6-7 discs was not significant (*p* > 0.05). No significant differences in individual disc internal pressures were observed between Neutral and Rotation-Anteflexion position (*p* > 0.05) (Figure 4, Table 1).

**TABLE 1:** Intradiscal Pressure in different conditions during Rotation-Traction Manipulation.

Condition	C2-3	C3-4	C4-5	C5-6	C6-7	C 2–7 mean
Nautral Position	0.84 (0.46)	0.76 (0.44)	0.44 (0.29)	0.48 (0.36)	0.43 (0.26)	0.74 (0.31)
Rotation-Anteflexion	0.91 (0.53)	0.92 (0.60)	0.57 (0.40)	0.57 (0.42)	0.59 (0.37)	0.88 (0.40)
Preload	0.30 (0.24)	0.31 (0.28)	0.10 (0.16)	0.09 (0.15)	0.10 (0.12)	0.19 (0.24)
Thrust	0.07 (0.16)	0.12 (0.19)	−0.10 (0.15)	−0.02 (0.13)	0.11 (0.08)	0.03 (0.17)

Data are expressed as Mean (SD). Cn-n represents the disc of the corresponding segment. C 2–7 Mean represents the average of intradiscal pressures in five intervertebral disc. The thrust parameters are chosen as 150N force and 0.1 s duration

**FIGURE 5:**
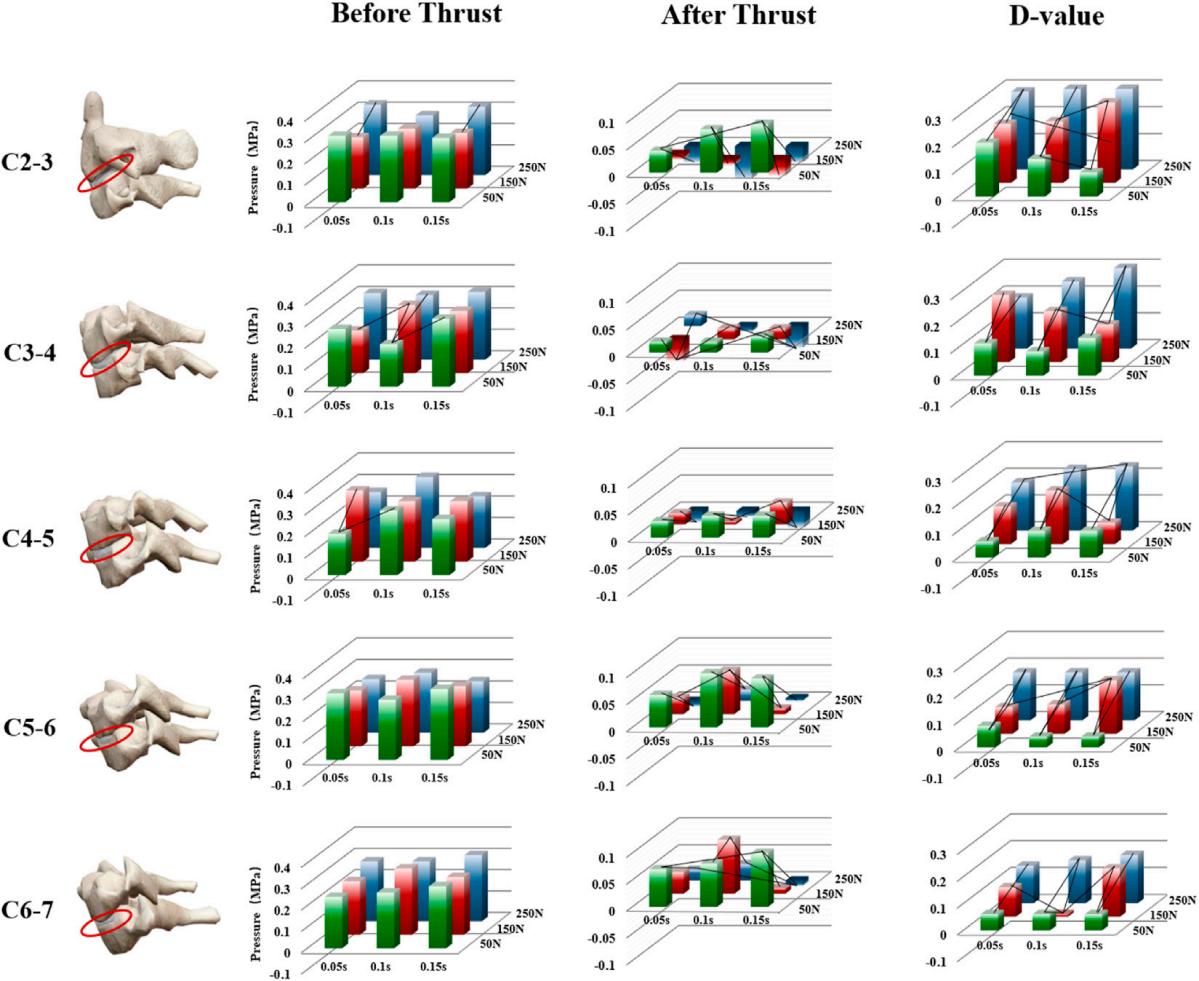
Variations in intradiscal pressure of different intervertebral discs during the application of manipulation. Statistically significant disparities in intranuclear pressure between different parameters (force and duration) are indicated by connecting lines. D-value: difference value of intradiscal pressure before and after manipulation.

Taking into account the different variations caused by preloading, we counted the difference value of Intradiscal pressure between Thrust and Preload (Figure 5). When the thrust duration was fixed, there was a significant difference of Intradiscal pressure changes between different thrust force (*p* < 0.05), and it was positively correlated with the magnitude of force. Further two-by-two comparison in LSD, 150 N thrust force shows a more significant decrease of Intradiskal pressure than 50 N (*p* < 0.05), and a decrease was also observed with 250 N–150 N despite this was not statistically significant (*p* > 0.05) (Table 2).

**TABLE 2:** Difference value of Intradiscal pressure between Thrust and Preload with different force and duration parameters.

Duration (s)	Thrust force (N)	C2-3	C3-4	C4-5	C5-6	C6-7	C 2-7 (mean)
0.05	50	0.20 (0.08)	0.12 (0.07)	0.06 (0.09)	0.08 (0.06)	0.06 (0.06)	0.08 (0.04)
	150	0.22 (0.13)	0.25 (0.10)	0.14 (0.12)	0.10 (0.08)	0.11 (0.06)	0.16 (0.06)
	250	0.29 (0.13)	0.19 (0.17)	0.18 (0.16)	0.18 (0.14)	0.14 (0.10)	0.18 (0.08)
0.1	50	0.14 (0.06)	0.09 (0.05)	0.10 (0.07)	0.04 (0.05)	0.06 (0.04)	0.08 (0.03)
	150	0.23 (0.08)	0.19 (0.09)	0.20 (0.06)	0.11 (0.06)	0.01 (0.04)	0.16 (0.07)
	250	0.31 (0.16)	0.25 (0.09)	0.23 (0.10)	0.18 (0.09)	0.16 (0.09)	0.21 (0.08)
0.15	50	0.09 (0.10)	0.14 (0.08)	0.10 (0.06)	0.04 (0.05)	0.06 (0.04)	0.09 (0.04)
	150	0.30 (0.17)	0.14 (0.09)	0.08 (0.12)	0.20 (0.15)	0.18 (0.14)	0.15 (0.06)
	250	0.33 (0.20)	0.24 (0.13)	0.15 (0.09)	0.18 (0.13)	0.18 (0.11)	0.17 (0.08)

Data are expressed as Mean (SD). Cn-n represents the disc of the corresponding segment. C 2–7 mean represents the average of intradiscal pressures in five intervertebral disc.

**FIGURE 6:**
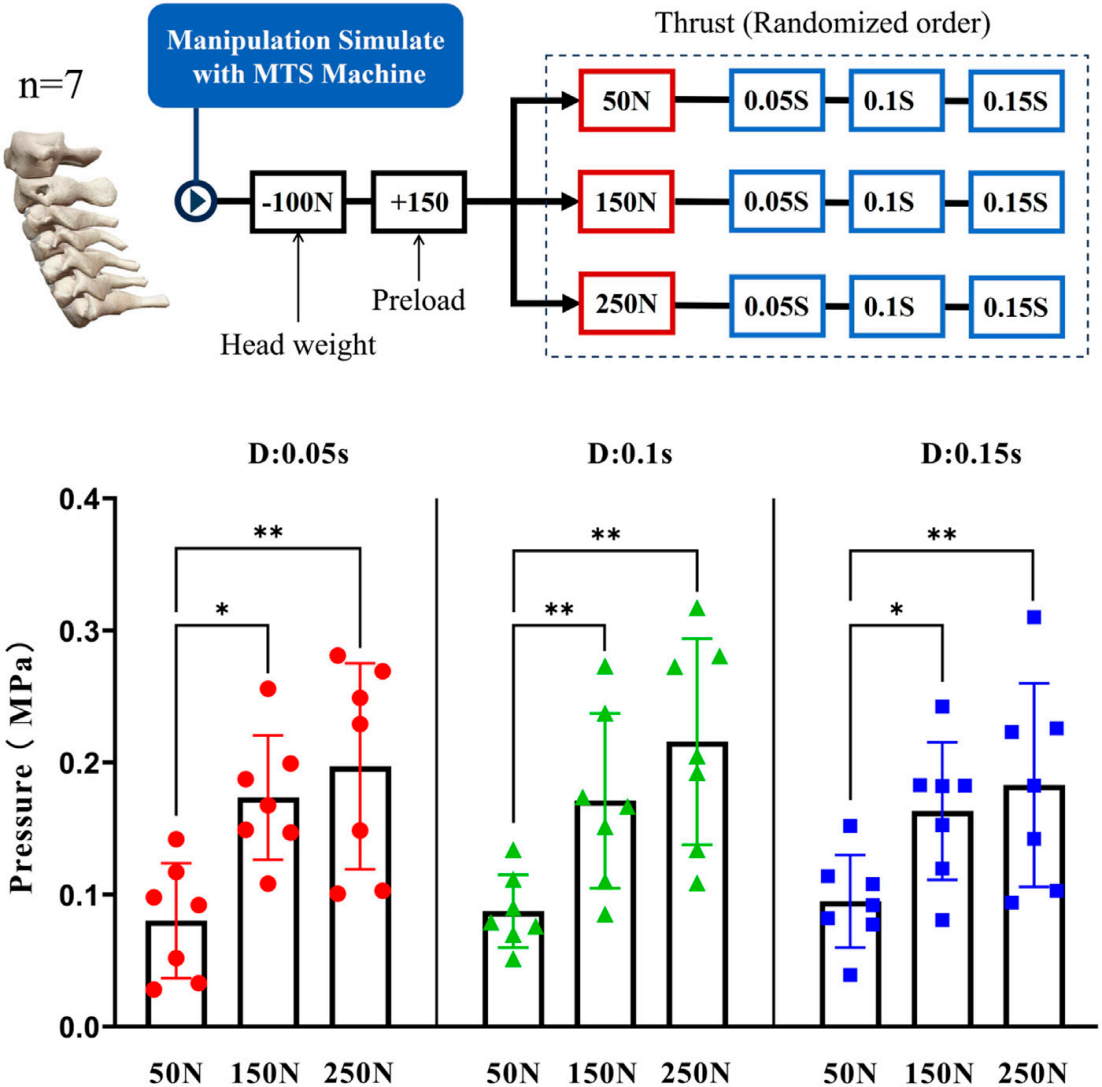
Depict the manipulation of cervical spine specimens using the MTS machine under varied mechanical and temporal parameters (above), and the loading sequence for different parameters was entirely randomized. Furthermore, a comparative analysis was conducted on the differences in intradiscal pressure between varying force magnitudes and durations (below), and the intradiscal pressure data were derived from the difference between Preload and Thrust.

When the thrust force was fixed, there was no significant difference of Intradiscal pressure changes between different thrust duration (*p* > 0.05). The Intradiskal pressure seemed to be unaffected regardless of the variation in duration (Figure 6).

### 3.2 Variation of intradiscal pressure during cervical traction

**FIGURE 7:**
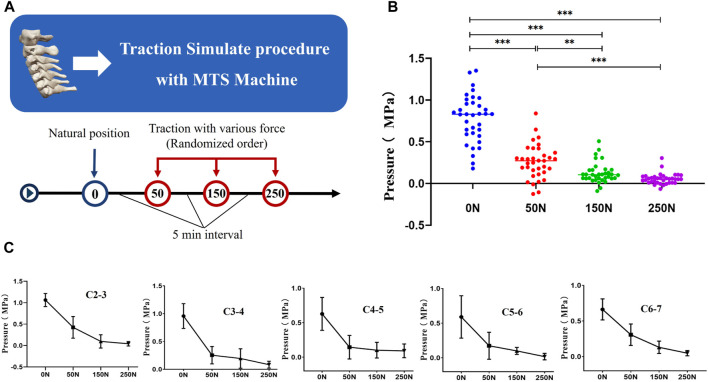
**(A)** The MTS machine simulates the process of cervical spine traction. **(B)** Examining the variations in intradiscal pressure among different traction forces. **(C)** Delving into the specific changes in nucleus pulposus pressure for each intervertebral disc.

The average intervertebral disc pressure in the neutral position was 0.78 (0.28) MPa, reduced to 0.26 (0.20) MPa under 50N traction, further decreased to 0.12 (0.12) MPa under 150N traction, and reached 0.05 (0.07) MPa under 250N traction. In all cases, intervertebral disc pressure significantly decreased post-traction (*p* < 0.05). We assessed the impact of traction force variations on intervertebral disc pressure, revealing statistically significant differences among different traction forces (*p* < 0.05), with a positive correlation observed between traction force magnitude and disc pressure reduction. Further pairwise comparisons using LSD indicated that 150N traction was more effective in significantly reducing intervertebral disc pressure compared to 50N traction (*p* < 0.05), and 250N traction demonstrated a more pronounced decrease in disc pressure compared to 150N traction (*p* < 0.05) (Figure 7, Table 3).

**TABLE 3:** Intradiscal Pressure in different conditions during Cervical Traction.

Traction force (N)	C2-3	C3-4	C4-5	C5-6	C6-7	C 2–7 mean
0	1.06 (0.15)	0.96 (0.22)	0.63 (0.24)	0.59 (0.31)	0.66 (0.15)	0.78 (0.28)
50	0.42 (0.25)	0.25 (0.16)	0.14 (0.17)	0.17 (0.19)	0.31 (0.15)	0.26 (0.20)
150	0.10 (0.15)	0.19 (0.17)	0.10 (0.11)	0.10 (0.05)	0.13 (0.09)	0.12 (0.12)
250	0.04 (0.05)	0.08 (0.06)	0.09 (0.06)	0.17 (0.05)	0.05 (0.04)	0.05 (0.07)

Data are expressed as Mean (SD). Cn-n represents the disc of the corresponding segment. C 2–7 mean represents the average of intradiscal pressures in five intervertebral disc.

### 3.3 Comparison of intradiscal pressure changes between RTM and cervical traction

**FIGURE 8:**
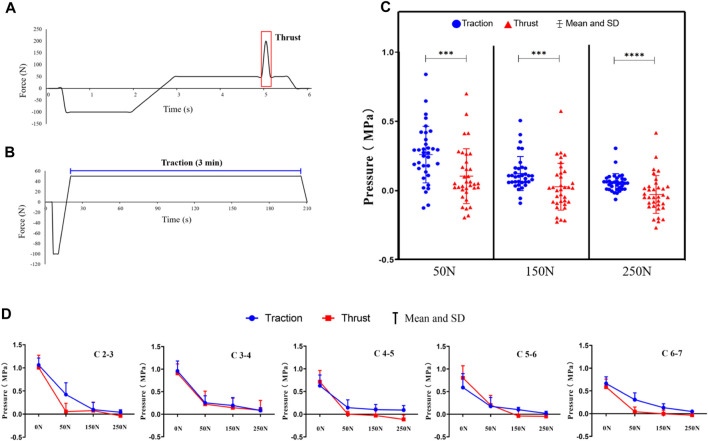
**(A)** Force-time graphs during the simulation of Rotation-Traction Manipulation using the MTS machine. **(B)** Force-time graphs during the simulation of cervical spine traction using the MTS machine. **(C)** A comparative analysis of the intradiscal pressure changes induced by Rotation-Traction Manipulation and cervical spine traction. **(D)** Specific comparisons of changes within each intervertebral disc. Note: data were collected at the points of traction cessation and thrust cessation.

We conducted calculations to assess the impact of variations in RTM and cervical spine traction on intervertebral disc pressure, followed by a comparative analysis. Irrespective of the magnitude of applied forces (50N, 150N, or 250N), we observed that manipulation was significantly more effective in reducing intervertebral disc pressure than sustained traction (*p* < 0.05). Data were collected at the points of traction cessation and thrust cessation, with the average values obtained for the intervertebral discs at C2-7 (Figure 8, Table 4).

**TABLE 4:** Intradiscal Pressure in different conditions during Rotation-Traction Manipulation.

Loading force (N)	Loading procedure		*t*	*P*
	Thrust	Traction		
50	0.10 (0.20)	0.26 (0.20)	3.46	0.001
150	0.03 (0.16)	0.14 (0.14)	3.679	0.001
250	−0.03 (0.13)	0.07 (0.08)	4.103	<0.001

Data are expressed as Mean (SD). Data were collected at the points of traction cessation and thrust cessation, with the average values obtained for the intervertebral discs at C2-7.

## 4 Discussion

This study aimed to describe the impact of cervical Rotation-Traction Manipulation (RTM) with varying levels of force and duration on the intradiscal pressure within human cervical spine specimens, and compare the impact on intradiscal pressure between RTM and traction. The research findings indicate that both RTM and traction significantly reduce the intradiscal pressure of the cervical spine. Moreover, the nucleus pulposus pressure decreases further with increasing force parameters (50N, 150N, 250N). However, the intradiscal pressure seems unaffected regardless of changes in the thrust duration (0.05 s, 0.1 s, 0.15 s). And a comparative analysis revealed that RTM more markedly reduced intradiscal pressure compared to traction alone.

The intervertebral disc occupies one-third of the spinal column’s height. Its primary function is to evenly distribute loads and allow for minor spinal movements (Holck, 2010; Nnwell et al., 2017; Rizwan et al., 2022). It forms a closed buffer system against gravity and tension, composed of the annulus fibrosus, nucleus pulposus, and cartilaginous endplates. The nucleus pulposus possesses excellent water-retaining capacity, creating a hydrostatic environment within. Its role is to evenly distribute stress to the annulus fibrosus, vertebral bodies, and the entire spine. Due to the tension exerted by the annulus fibrosus and the muscles and ligaments surrounding the intervertebral disc, the nucleus pulposus remains in a preloaded state, aiming to maintain intrinsic stability of the spine (Fearing et al., 2018; Li et al., 2022; Andrea et al., 2023). Existing research supports that decompression of the intervertebral disc may alleviate discogenic pain associated with intervertebral disc degeneration by reducing articular surface load and nerve root compression (Pickar, 2002; Bialosky et al., 2008). Therefore, measuring changes in intranuclear pressure during traction maneuvers can effectively reflect the mechanical environment within the cervical spine and potential biomechanical mechanisms.

In the realm of measuring internal pressures within intervertebral discs, various techniques have been employed. Kramer (1977) involve direct measurement inside lumbar intervertebral discs of lucid volunteers using a pressure-measuring needle setup, was the most widely used method. However, this method presents limitations when it comes to assessing the internal pressures of dynamic cervical discs subjected to rotational movements. One significant challenge stems from the slender nature of cervical discs in human, with an average front edge height of merely 4.37 mm (Pelker et al., 1991). This poses difficulty for pressure-measuring needles with a diameter of 1.3 mm to puncture the disc, leading to potential damage, causing at least 25% sectioning injuries to the anulus fibrosus. Furthermore, during cervical spine rotation, shearing forces generated by the anulus fibrosus may jeopardize the integrity of the pressure-measuring needles and signal transmission.

To address these limitations, this experiment draws inspiration from and enhances the measuring method pioneered by P. A. Cripton (Miura et al., 2002). It utilizes five micro pressure sensors, allowing for simultaneous measurement of internal pressures within the nucleus pulposus of intervertebral discs spanning C2 to C7. This method capitalizes on the use of small and delicate sensors, minimizing potential harm to the anulus fibrosus and nucleus pulposus during the puncturing process. Once implanted within the nucleus pulposus, only three fine electric wires remain within the anulus fibrosus, ensuring that even substantial cervical spine movements, including rotation, bending, and stretching, do not compromise structural integrity. Notably, this approach permits direct and dynamic measurement of nucleus pulposus internal pressures with exceptional sensitivity. Additionally, the equipment used for pressure signal collection is straightforward and convenient, enabling the measurement of internal pressures across multiple nucleus pulposus of intervertebral discs, surpassing previous methods limited to single-disc pressure measurements. Remarkably, the static internal pressure values measured in cervical nucleus pulposus closely align with those reported by P. A. Cripton.

In this experimental context, adult cervical spine specimens of slightly advanced age were selected. As indicated by X-ray imaging, these specimens exhibited varying degrees of retrogressive changes. These changes, attributed to fibrosis-induced dehydration, likely resulted in differing levels of decreased internal pressures within the retrograded nucleus pulposus (Ferguson and Steffen, 2003; Park et al., 2005). It's worth noting that these discrepancies in internal pressures between normal and degraded intervertebral discs could potentially introduce variations in the statistical analysis of internal pressures within different sections of the nucleus pulposus when subjected to the same load (VELÍSKOVÁ et al., 2018), as well as in their observed trends. While clinical diagnosis and classification of intervertebral disc degeneration often utilize MRI scan (Zhang et al., 2022; Liawrungrueang et al., 2023), this approach is not applicable to *in vitro* specimens. We observed that such artificially induced observations were prone to substantial subjective deviations. Moreover, due to the limited number of specimens available for this study, we were unable to carry out a comprehensive layer-by-layer analysis of the experimental results.

After subjecting the cervical spine to various RTM in different states, a consistent observation emerged—there was a discernible decrease in the internal pressures of the cervical nucleus pulposus. This decrease in pressure became more pronounced as the applied lifting force increased. This phenomenon is corroborated by findings from a study involving experiments on fresh cadaveric specimens (Herzog, 2010). In this experiment, the 250N manipulation simulated the accumulation of a pre-traction force of 150N, ultimately reaching a stretching force of 400N, which is in close proximity to the critical threshold associated with parenchymal damage. The data obtained from this experimental setup reveal a noteworthy trend: as the upward pulling force approaches 400N, the decline in internal pressure values of the cervical nucleus pulposus becomes less substantial, accompanied by a reduction in standard deviation. This nonlinear behavior indirectly suggests that the traction force has either neared or reached the critical point for cervical spine instability. Consequently, in clinical practice, it is prudent to apply a pre-traction force of 150N, which has proven effective in safely decreasing the internal pressures of the cervical nucleus pulposus. It is essential to exercise caution when lifting and thrusting with force exceeding 250N after applying 150N of pre-traction, as doing so may risk causing injury to the cervical spine. Vigorous manipulations of this nature should be avoided in clinical settings at all costs.

This study has several limitations. Firstly, The intervertebral discs and nucleus pulposus structures in cadaveric specimens exhibit certain disparities from their physiological states. It is imperative to approach the results with heightened caution. Secondly, to preserve the integrity of nucleus pulposus, only a single sensor was inserted into each nucleus pulposus. The choice between precise microdamage measurement and comprehensive multi-site damage measurement poses a conflicting dilemma. Thirdly, The intradiskal pressure measurement tool employed in this study requires the specimens to be maintained in a stable condition, making it challenging to capture the dynamic changes in intradiskal pressure throughout the entire operative procedure. Finally, this study primarily offers valuable insights and potential biomechanical explanations for the manipulation mechanism. However, as a comprehensive intervention, its clinical efficacy necessitates consideration from multiple perspectives.

## Data Availability

The original contributions presented in the study are included in the article/Supplementary material, further inquiries can be directed to the corresponding authors.
